# Metabolome and molecular basis for carbohydrate increase and nitrate reduction in burley tobacco seedlings by glycerol through upregulating carbon and nitrogen metabolism

**DOI:** 10.1038/s41598-018-31432-3

**Published:** 2018-09-05

**Authors:** Yafei Li, Dong Chang, Huijuan Yang, Jing Wang, Hongzhi Shi

**Affiliations:** 1 National Tobacco Cultivation & Physiology & Biochemistry Research Center, Henan Agricultural University, 450002 Zhengzhou, China; 2 Pingdingshan Branch of Henan Provincial Tobacco Company, 467002 Pingdingshan, China

## Abstract

Burley tobacco (*Nicotiana Tabacum*) is a chlorophyll-deficiency mutant. Nitrate is one precursor of tobacco-specific nitrosamines (TSNAs) and is largely accumulated in burley tobacco. To decrease nitrate accumulation in burley tobacco, glycerol, a polyhydric alcohol compound and physiological regulating material, was sprayed and its effects were investigated based on metabolomic technology and molecular biology. The results showed that glucose, glutamine and glutamic acid increased by 2.6, 5.1 and 196, folds, respectively, in tobacco leaves after glycerol application. Nitrate content was significantly decreased by 12–16% and expression of eight genes responsible for carbon and nitrogen metabolism were up-regulated with glycerol applications under both normal and 20% reduced nitrogen levels (*P* < 0.01). Leaf biomass of plants sprayed with glycerol and 20% nitrogen reduction was equivalent to that of no glycerol control with normal nitrogen application. Carbohydrates biosynthesis, nitrate transport and nitrate assimilation were enhanced in glycerol sprayed burley tobacco seedlings which might contribute to reduced nitrate and increased carbohydrates contents. In conclusion, glyerol spray coupled with 20% nitrogen reduction would be an effective method to reduce nitrate accumulation in burley tobacco.

## Introduction

Burley tobacco is one type of chlorophyll deficiency, and is well known as yellow-green leaf tobacco with reduced chlorophyll content and lower carbohydrate accumulation^1,2^. This mutations is correlated with one double homozygous recessive genotypes at *Yellow Burley 1* (*YB1*) and *Yellow Burley 2* (*YB2*) loci, and has been reported that the changes resulted in low nitrogen use efficiency and high levels of nitrate accumulation which is one of the important precursors of tobacco-specific nitrosamines (TSNAs)^3,4^. TSNAs are easy to induce malignant tumors in animals and was classified as group 1 carcinogens by International Agency for Research on Cancer^5^. Nitrate was greater in burley tobacco than in other tobacco types^6^. Nitrate is mainly accumulated during plant growth and development in the field^7^. Mostly, the low capacity of nitrogen assimilation and translocation lead to nitrate accumulation in burley tobacco^8^. Moreover, decreasing nitrate content in burley tobacco is always the important approach to lower the formation of TSNAs. There were limited reports related to strategies aiming at nitrate reduction during the process of tobacco growth and development.

In general, nitrogen metabolism is highly correlated with carbon metabolism in plants. Photosynthesis carbohydrates provide carbon backbones for nitrogen assimilation^9^. Most enzymes involved in nitrogen assimilation require reducing powers for their reactions in chloroplasts, and their activities are highly regulated by photosynthesis and its products^10^. Glycerol, which can supply carbon atom in conditions of reduced photosynthesis carbon fixation in plant^11^, was involved in cell growth, maintenance and metabolism^12^. Glycerol can freely inter into cells through water channels^13^. It is reported that glycerol is transported across the plasma membrane via MIPs/aquaporins^14^. Glycerol played an important role in cellular osmoregulation, photosynthetic carbon metabolism and starch metabolism^15,16^, and it can be converted to sucrose by glycolysis and gluconeogenesis^17^. There are two pathways reportedly related to glycerol metabolism in plant^18^. The first one, glycerol is phosphorylated to glycerol-3-phosphate (G-3-P) by glycerol kinase (EC 2.7.1.30) and then G-3-P is catalyzed to dihydroxyacetone phosphate (DHAP) by a G-3-P dehydrogenase (GPDH)^19^. In this pathway, the enzyme of glycerol kinase plays a rate-limiting role in glycerol metabolism^20,21^. In the second pathway, glycerol is converted to dihydroxyacetone by NAD^+^-glycerol dehydrogenase (EC 1.1.1.6) and is then phosphorylated to DHAP by dihydroxyacetone kinase (EC 2.7.1.29)^22^, and DHAP is involved in the process of glycolysis and gluconeogenesis for sugar production. In preliminary work, spraying glycerol had largely decreased nitrate accumulation in vegetables but effects and its mechanisms were not clearly demonstrated^23^. The hypotheses of reducing nitrate in burley tobacco by glycerol were conceived: (1) Burley tobacco with low chlorophyll content and carbohydrates formation is one chlorophyll deficiency mutant, and carbon skeleton and energy might be insufficient for nitrate reducing and assimilation; (2) Glycerol can be converted to sucrose through the glycolysis and gluconeogenesis, which might increase carbohydrates formation and provide more carbon skeleton and energy supply; (3) Nitrate transport might be induced by sufficient energy and nitrate assimilation be promoted, leading to decrease of nitrate storage in tobacco leaves.

In burley tobacco, high nitrate accumulation is correlated with nitrogen amount applied and reducing nitrogen level was effective in decreasing nitrate content^3^, although there is currently no in-depth coverage at molecular level in burley tobacco. From our early study, we have found that nitrogen application was 5-folds higher in burley tobacco than in flue-cured tobacco to produce similar leaf biomass, and lower contents of pigment and energy substances and weaker nitrogen assimilation were the key factors contributing to nitrate accumulation in burley tobacco^7,8^. In this study, glycerol was applied to regulate carbohydrate and nitrate accumulation in two burley tobacco varieties. RNA sequencing technology was used to analyze the effects of reducing nitrogen fertilizer level on metabolism in burley tobacco seedlings, and metabolome analysis was used to explore the regulating effects of exogenous glycerol on carbohydrate biosynthesis and nitrate reduction in burley tobacco seedlings. Pigment content, photosynthetic trait, nitrate and carbohydrates content, expression of genes related to carbon and nitrogen assimilation and glycerol metabolism were investigated at sufficient and 20% reducing nitrogen applying levels, aiming to provide effective methods to decrease nitrate accumulation and TSNAs formation in burley tobacco cultivation.

## Results

### RNA-Seq Statistics, and analysis of DEGs induced by deficient nitrogen

**Figure 1:**
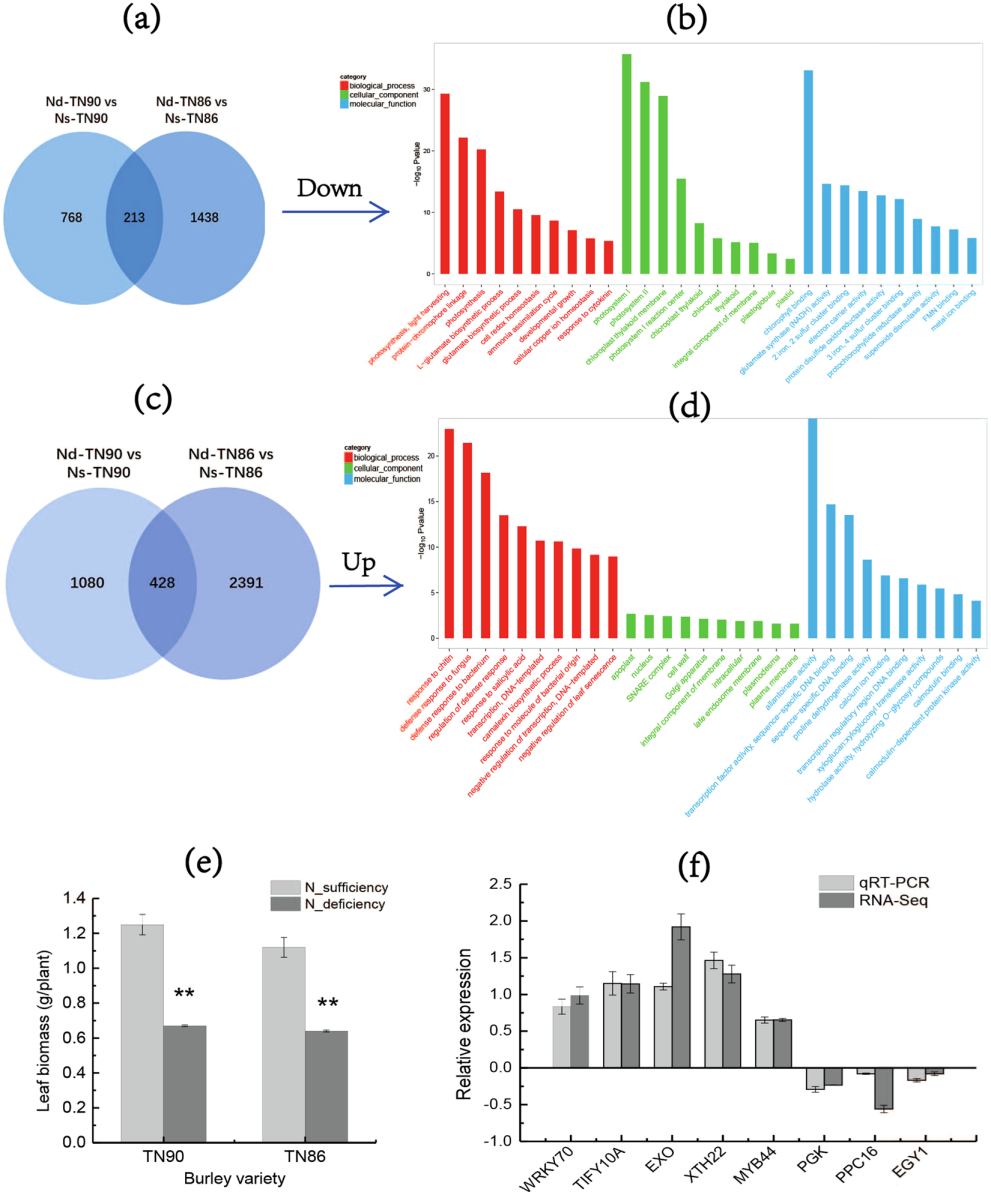
Transcriptome analysis strategies and GO enrichment of target DEGs and leaf biomass (**a**) Venn diagram of down-regulated DEGs of deficient nitrogen condition vs sufficient nitrogen condition in TN90 and TN86 (**b**) GO enrichment in common down-regulated DEGs between TN90 and TN86 (**c**) Venn diagram of up-regulated DEGs of deficient nitrogen condition vs sufficient nitrogen condition in TN90 and TN86 (**d**) GO enrichment in common up-regulated DEGs between TN90 and TN86 (**e**) Leaf biomass (**f**) RNA-seq results confirmed by quantitative qRT-PCR. Nd-TN90: TN90 cultivated under deficient nitrogen condition; Ns-TN90: TN90 cultivated under sufficient nitrogen condition; Nd-TN86: TN86 cultivated under deficient nitrogen condition; Ns-TN86: TN86 cultivated under sufficient nitrogen condition. Error bars of leaf biomass indicate standard error of the means (n = 3). Error bars of qRT-PCR indicate standard error of the means (n = 6).

In this study, leaf biomass was significantly lowered for low nitrogen plants than that for normal nitrogen plants (*P* < 0.01, Fig. 1e). There were twelve cDNA libraries (2 varieties * 2 nitrogen application levels * 3 biological replicates) prepared to analyze the effects of nitrogen deficiency on metabolism in burley tobacco seedlings. After removing sequencing adaptors and low quality reads, 72.56 M reads were obtained in tobacco leaves under nitrogen sufficiency and nitrogen deficiency conditions. A total of 85.14% reads from TN90 and 84.82% reads from TN86 were mapped to the reference genome with almost 81% of them having unique location in that genome.

The DEGs induced by nitrogen deficiency both in TN90 and TN86 at the same time were highly obtained. In all, 428 of up-regulated common genes and 213 of down-regulated common genes were analyzed, respectively (Fig. 1a,c). There are 12.85% of up-regulated common genes involved in the biological process of defense response (GO:0050832, GO:0042742, GO:0031347) (Fig. 1d). There are almost 15.96% of down-regulated common genes correlated with biological process (GO-BP) of photosynthesis (GO:0009765, GO:0015979). We observed high values for categories involved in gene ontology cellular component (GO-CC), such as photosystem I (GO:0009522), photosystem II (GO:0009523), chloroplast thylakoid membrane (GO:0009535), cytosol (GO:0005829). Genes mostly involved in gene ontology molecular function (GO-MF), such as chlorophyll binding (GO:0016168), glutamate synthase (NADH) activity (GO:0016040).

qRT-PCR analysis of 8 DEGs was conducted to validate the accuracy of RNA-Seq date. Expression patterns of selected genes were consistent with those in RNA-seq assay, indicating that results of RNA-Seq were reliable.

### Differences of metabolites determined by GC/MS

**Figure 2:**
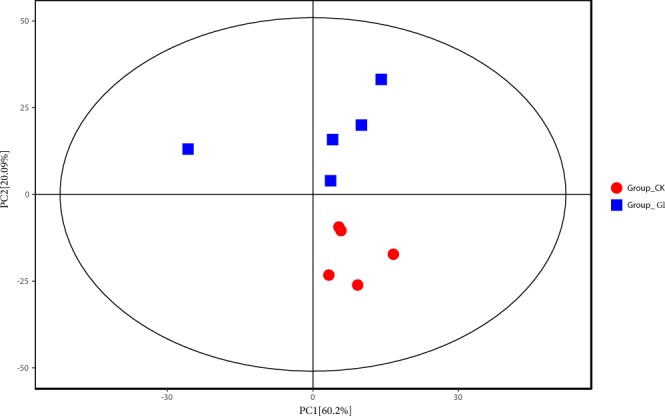
PCA of metabolome of spraying glycerol at normal nitrogen level. The blue boxes indicate that control group with no spraying glycerol. The red circles indicate that treatments with spaying glycerol.

Fifteen metabolites determined by metabolome between treatments were different, including amino acids and carbohydrates (Table 1). The result of PCA was showed that differences between treatments were substantial (Fig. 2). Amino acids and carbohydrates content were increased by spraying glycerol, including glucose, sucrose, fructose-6-phosphate, succinic acid and fumaric acid, which were mainly correlated with metabolic pathway of glycolysis, TCA cycle and starch and sucrose metabolism. Glutamine and L-glutamic acid were also significantly increased by spraying glycerol (*P* < 0.01).

**Table 1:** Profiles of metabolites in response to glycerol spray on burley tobacco seedling leaf under sufficient nitrogen condition.

Pathway	Name	Fold Change	P-Value
Nitrogen metabolism	glutamine	3.6	0.003
	glutamic acid	6.0	0.002
Porphyrin and chlorophyll metabolism	glycine	1.6	0.011
Carbon fixation in photosynthetic organisms	sedoheptulose	1.5	0.017
Glycolysis, TCA cycle	fructose-6-phosphate	2.4	0.014
	succinic acid	1.6	0.010
	fumaric acid	1.5	0.031
	glycerol	1.84	0.010
Starch and sucrose metabolism	glucose	200	0.001
	glucuronic acid	36	0.010
	sucrose	1.4	0.009
	trehalose-6-phosphate	1.4	0.112
	cellobiose	1.3	0.160
	maltose	1.2	0.329
	D-galacturonic acid	1.2	0.341

Fold Change: Ratio of glycerol treatment to the control.

### Expression levels of genes induced by glycerol

**Figure 3:**
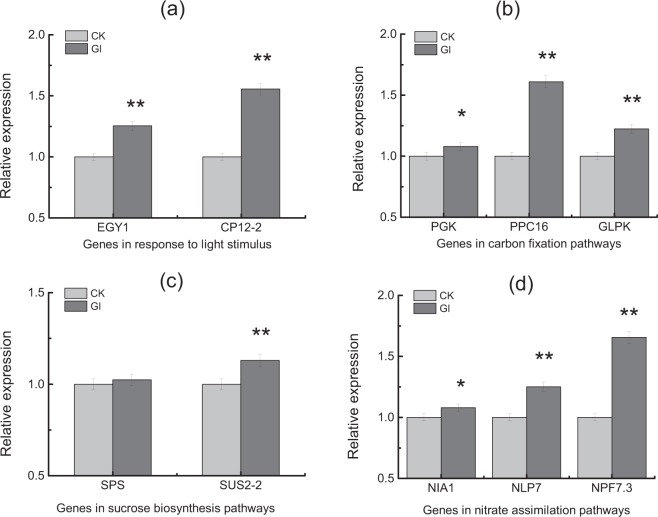
Light stimulus, carbon fixation, sucrose biosynthesis and nitrate assimilation induced by spraying glycerol at 2 days after spray. Expression levels of ten genes were obtained by qRT-PCR. (**a**) Genes in response to light stimulus. (**b**) Genes in carbon fixation pathways (**c**) Genes in sucrose biosynthesis pathways. (**d**) Genes in nitrate assimilation pathways. CK: control; Gl: glycerol. *EGY1* (Probable zinc metalloprotease, Gene ID 833476), *CP12-2* (Calvin cycle protein, Gene ID 825414), *PGK* (Phosphoglycerate kinase, Gene ID 107787830), *PPC16* (Phosphoenolpyruvate carboxylase, Gene ID 547769), *GLPK* (glycerol kinase [Nicotiana attenuata], Gene ID 109221820), *SPS* (sucrose-phosphate synthase, Gene ID 107766133), *SUS2_2* (sucrose synthase [Nicotiana attenuata], Gene ID 109214879), *NIA1* (nitrate reductase [NADH] 1 [Nicotiana attenuata], Gene ID 107823732), *NLP7* (Protein NLP7, Gene ID 828502), *NPF7*.*3* (Protein NRT1/ PTR family 7.3, Gene ID 840139). Error bars of qRT-PCR indicate standard error of the means (n = 3). Symbols **and * indicate that the significant differences between treatment of control and treatment of spraying glycerol are at 0.01 and 0.05, respectively.

Two days after spraying glycerol, expression of genes involved into response to light stimulus (*EGY1*, *CP12-2*), carbon fixation pathways (*PGK*, *PPC16*, *GLPK*), sucrose biosynthesis (*SPS*, *SUS2-2*), nitrate transport and nitrate assimilation (*NIA1*, *NLP7*, *NPF7*.*3*) were all up-regulated (Fig. 3). Compared to CK, expression of genes *CP12-2*, *PPC16*, *NPF7*.*3* increased more than 0.5-fold after spraying glycerol, indicating that glycerol might enhance carbon fixation, photosynthesis and nitrate transport in tobacco leaves.

**Figure 4:**
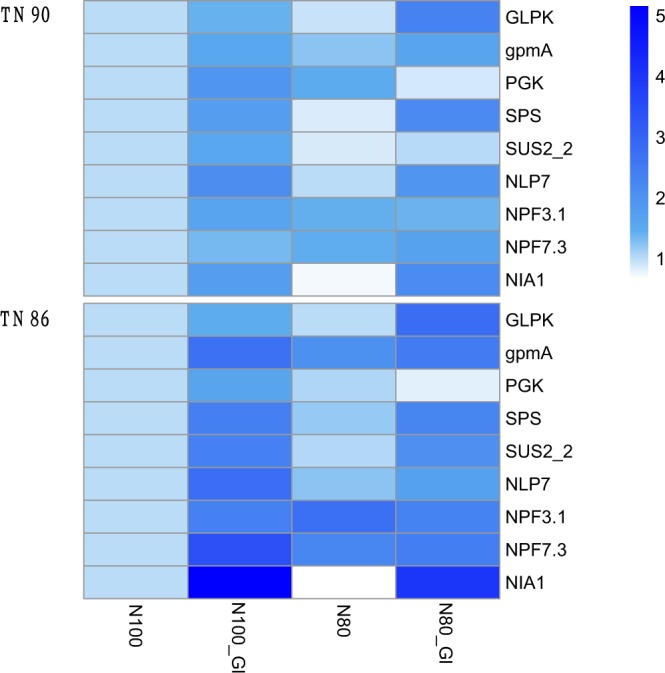
Glycerol enhanced carbon and nitrogen metabolism in burley tobacco 7 days after spray under different nitrogen levels. Expression levels of nine genes were quantified by qRT-PCR. Carbon metabolism: *GLPK* (glycerol kinase [Nicotiana attenuata], Gene ID 109221820), *gpmA* (2,3-bisphosphoglycerate-dependent phosphoglycerate mutase, accession number BX908798), *PGK* (Phosphoglycerate kinase, Gene ID 107787830), *SPS* (sucrose-phosphate synthase, Gene ID 107766133), *SUS2_2* (sucrose synthase [Nicotiana attenuata], Gene ID 109214879). Nitrogen metabolism: *NLP7* (Protein NLP7, Gene ID 828502), *NPF3*.*1* (Protein NRT1/ PTR family 3.1, Gene ID 843186), *NPF7*.*3* (Protein NRT1/ PTR family 7.3, Gene ID 840139), *NIA1* (nitrate reductase [NADH] 1 [Nicotiana attenuata], Gene ID 107823732).

Seven days after spraying glycerol, differences in genes expression levels related to carbohydrates biosynthesis and nitrate assimilation between treatments were all substantial under both sufficient and 20% nitrogen reduction conditions. And expression of genes (*SUS2-2*, *SPS*) involved in glucose and starch synthesis were all up-regulated (Fig. 4), which was consist with the increase of carbohydrate in burley tobacco seedlings. Expression of genes *GLPK*, *gpmA* and *PGK* correlated with glycerol metabolism were up-regulated by spraying glycerol under different nitrogen conditions. After spraying glycerol, changes of genes expression levels were greater at 7 days after than at 2 days after. Glycerol induced the biosynthesis of carbohydrates and chlorophyll, which would lead to increase of carbohydrate contents and biomass accumulation in burley tobacco seedlings. Moreover, expression of genes (*NLP7*, *NIA1*, *NPF3*.*1* and *NPF7*.*3*) correlated with nitrate transport and assimilation were all up-regulated by glycerol.

### Increase of chlorophyll and carbohydrate formation by glycerol

**Figure 5:**
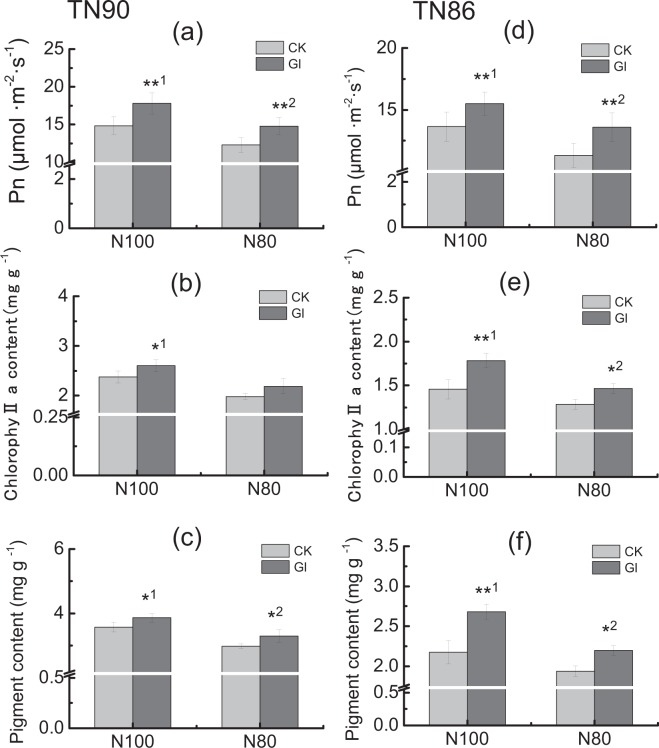
Effect of pigment content and photosynthesis rate by spraying glycerol under different nitrogen conditions (**a**,**d**) Pn (**b**,**e**) Chlorophyll a (**c**,**f**): Pigment. CK: control; Gl: glycerol. Pn: photosynthesis rate. Error bars of photosynthesis rate indicate standard error of the means (N = 6, “N” means the number of individuals), and error bars of chlorophyll a content and pigment content indicate standard error of the means (n = 3, three biological replicates). Symbols ** ^1^and * ^1^indicate that the significant differences between treatment of control and treatment of spraying glycerol under sufficient nitrogen condition (N100) are at 0.01 and 0.05, respectively. Symbols ** ^2^and * ^2^indicate that the significant differences between treatment of control and treatment of spraying glycerol under 20% less nitrogen application condition (N80) are at 0.01and 0.05, respectively (**a**–**c**) TN90 (**d**–**f**), TN86.

**Figure 6:**
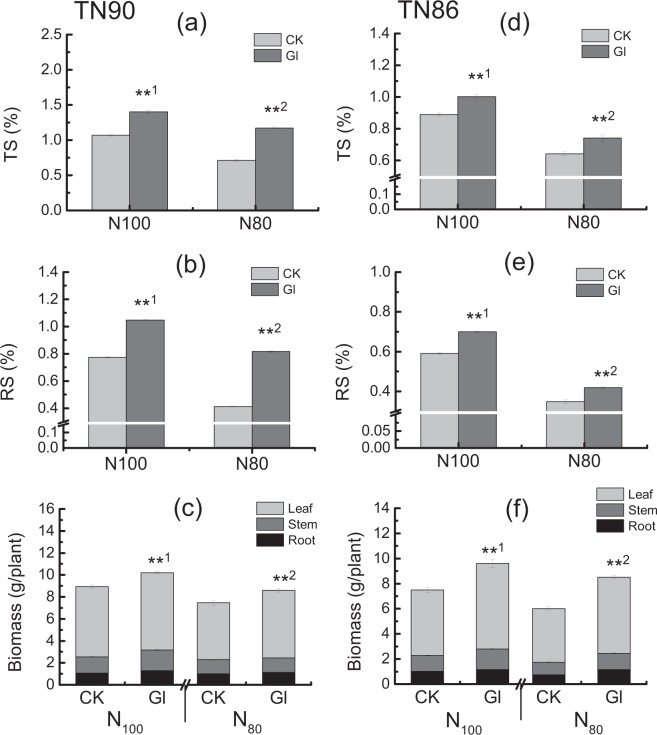
Effect of spraying glycerol on total soluble sugar (**a,d**), reducing sugar content (**b,e**) and biomass accumulation (**c,f**) in burley tobacco seedlings under different nitrogen conditions. CK: control; Gl: glycerol. TS: total soluble sugar content. RS: reducing sugar content. Error bars indicate standard error of the means (n = 3). Symbols ** ^1^indicate that the significant differences between treatment of control and treatment of spraying glycerol under sufficient nitrogen application (N100) are at 0.01. Symbols ** ^2^indicate that the significant differences between treatment of control and treatment of spraying glycerol under 20% less nitrogen application condition (N80) are at 0.01 (**a**–**c**) TN90 (**d**–**f**) TN86.

Differences in leaf biomass, parameters of carbon and nitrogen metabolism and its products between varieties, treatments and their interactions were substantial in tobacco leaves (Table 2). Leaf biomass, chlorophyll a contents and photosynthetic rates in both TN90 and TN86 significantly increased by spraying glycerol under both normal and reduced nitrogen conditions, respectively (*P* < 0.05, Figs 5 and 6). It is noteworthy that the photosynthetic rate and contents of chlorophyll a and other pigments, leaf biomass in glycerol-sprayed plants with 20% less nitrogen application were all equivalent to those in no glycerol-sprayed plants with normal nitrogen application. The total sugar, soluble reducing sugar contents and biomass accumulation (root, stem, leaf) in both TN90 and TN86 significantly increased by glycerol treatment under two different nitrogen conditions, respectively (*P* < 0.01, Fig. 6).

**Table 2:** ANOVA analysis of differences between treatments, varieties and their interactions on LB, TN, SP, NO_3_-N, Pigment, Pn, TS and RS content in burley tobacco seedlings.

Parameters	LB	TN	SP	NO_3_-N	Chlorophyll a	Pigment	Pn	TS	RS
Varieties (V)	2.8	3.5	6.8	18.6	1.5	1.0	0.8	2.0	1.0
	ns	**	**	**	**	**	*	**	**
Treatments (T)	25.1	8.0	4.5	1.9	1.7	1.6	2.3	9.7	10.5
	**	**	*	ns	ns	ns	ns	**	**
Varieties (V)*Treatments (T)	67.8	201.9	40.7	1272.0	70.6	96.3	18.5	1244.1	3486.5
	**	**	**	**	**	**	**	**	**

Note: LB, Leaf biomass; TN, Total nitrogen content; SP, Soluble protein content; NO_3_-N, NO_3_-N content; TS, Total soluble sugar content; RS, Reducing soluble sugar content. Symbols ∗∗ and ∗ indicate significant difference at 0.01 or 0.05, respectively, and ns indicate that there was no significant difference between treatments.

### Reduction of nitrate and increase of soluble protein by glycerol application

**Figure 7:**
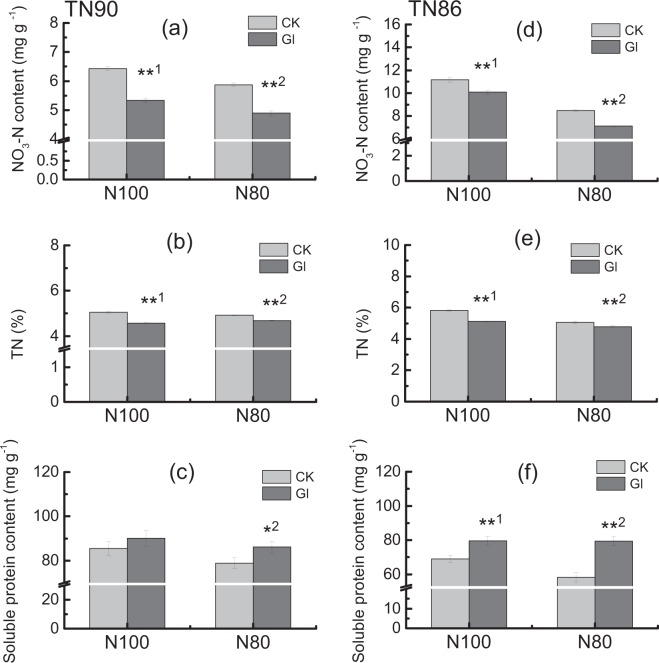
Effect of spraying glycerol on NO_3_-N (**a**,**d**), total nitrogen content (**b**,**e**) and soluble protein content (**c**,**f**) in burley tobacco seedlings under different nitrogen conditions. CK: control; Gl: glycerol. TN: total nitrogen content. Error bars indicate standard error of the means (n = 3). Symbols ** ^1^indicate that the significant differences between treatment of control and treatment of spraying glycerol under sufficient nitrogen condition (N100) are at 0.01. Symbols ** ^2^and * ^2^indicate that the significant differences between treatment of control and treatment of spraying glycerol under 20% less nitrogen application condition (N80) are at 0.01 and 0.05, respectively. (**a**–**c**), TN90; (**d**–**f**), TN86.

Total nitrogen and NO_3_-N content were significantly reduced by spaying glycerol under different nitrogen conditions in burley tobacco seedlings (*P* < 0.01, Fig. 7). In addition, soluble protein content in burley tobacco seedlings significantly increased after spaying glycerol at different nitrogen levels, indicating that the ability of nitrate reduction and assimilation in burley tobacco seedlings were enhanced by glycerol application (*P* < 0.05). Moreover, soluble protein content in glycerol-sprayed plants under 20% less nitrogen application condition was similar to the normal levels, while the NO_3_-N content was low.

## Discussion

Burley tobacco is one typical yellow-green leaf tobacco due to chlorophyll mutation, and chlorophyll and carbohydrate content are always lower than other tobacco types during the whole period of growth^24^. Nitrogen fertilizer application in burley tobacco is normally 4–5 fold higher than other tobacco types to produce the same levels of biomass, which resulted in low nitrogen use efficiency and more than 100 times higher of nitrate content in burley tobacco, further contributing to much more TSNA formation during both leaf curing and storage^2,3^. Transcriptomic analysis showed that expression of genes involved in photosynthesis were decreased by reducing nitrogen application, resulting in decreased leaf biomass accumulation and carbohydrates formation in burley tobacco^25^. Photosynthesis rate and leaf biomass accumulation for the treatment of 20% nitrogen reduction coupled with glycerol spray were similar to those in the control with sufficient nitrogen application, representing that glycerol can compensate one part of nitrogen fertilizer reduction in burley tobacco cultivation.

In burley tobacco, we found that low pigment and carbohydrates and weak ability of nitrogen assimilation were the key factors contributing to nitrate accumulation^7,8^. And nitrate concentration can be diluted with increased biomass in plant at the same nitrogen level^7^. In this work, spraying glycerol decreased nitrate accumulation in burley tobacco seedlings. For a better understanding of glycerol application in reducing nitrate and rising carbohydrates, technologies of molecular biology combined with metabolomes were used to explore the molecular mechanisms. Spraying glycerol has increased expression of genes involving into response to light stimulus, carbon fixation, nitrate transport and assimilation, and photosynthesis rate, nitrate assimilation and transportation abilities, and carbohydrates biosynthesis (glucose, starch and sucrose) were greatly induced in two burley tobacco varieties under different nitrogen conditions, indicating that spraying glycerol might enhance the ability of carbon and nitrogen assimilation in burley tobacco seedlings.

After spraying glycerol, glycerol metabolism was induced, which increased carbohydrates formation in burley tobacco seedlings. Glycerol is catalyzed to glycerol-3-phosphate (G-3P) by glycerol kinase (encoded by *GLPK* gene) and gene *GLPK* was significantly induced by spraying glycerol (*P* < 0.01). Glycerol-3-phosphate (G-3P) is one key crossroads of glucose, lipid and energy metabolism^26^. The 2, 3-bisphosphoglycerate-dependent phosphoglycerate mutase (EC 5.4.2.1, encoded by *gpmA* gene) and phosphoglycerate kinase (EC 2.7.2.3, encoded by *PGK* gene) can catalyze the interconversion of 3-phosphoglycerate (3-PGA) and 2-phosphoglycerate (2-PGA) during glycolysis and gluconeogenesis and are involved in photosynthetic carbon fixation^27^. Expression of genes *GLPK*, *PGK* and *gpmA* were significantly up-regulated by spraying glycerol under sufficient and 20% reducing nitrogen applying conditions, indicating that glycerol metabolism was greatly induced in burley tobacco seedlings (*P* < 0.05). In addition, genes related to starch and sucrose biosynthesis were also up-regulated after spraying glycerol, including *SPS*, which catalyzes the limiting steps in sucrose synthesis^28^, *SUS2-2*, which plays a dominant role in generating precursors for starch biosynthesis^29,30^. In analysis of metabolome, glucose, glucuronic acid and fructose-6-phosphate were markedly increased, which was consistent with increased expression of genes involved in carbohydrates biosynthesis.

Carbon metabolism supplies carbon skeleton and energy for nitrogen metabolism^31,32^. Nitrate is largely accumulated and carbohydrates are extremely low in burley tobacco, which are associated with one double recessive genes mutation^2^. In this study, nitrate content was significantly decreased and leaf biomass was significantly increased by spraying glycerol (*P* < 0.01), and the effect of 20% nitrogen reduction with spraying glycerol was the most effective in decreasing nitrate content in burley tobacco seedlings. Transcription factor *NLP7* is a positive regulator of nitrate-induced expression of N-related genes through post-translation regulation, such as genes *NRT2*.*1*, *NITR2:1*, *NIA1*, *NIR1*, which plays an important role in nitrate assimilation pathway^33–35^. *NLP7* was up-regulated by spraying glycerol, which was conducive to decreasing nitrate accumulation in burley tobacco leaves. Nitrate reductase (EC 1.6.6.1, encoded by *NIA* genes) catalyzes the limiting-rate step of nitrate reduction and assimilation in most organisms^36^. Expression of gene *NIA1* was up-regulated and the products of nitrogen assimilation (glutamic acid, glutamine and soluble protein) were increased by spraying glycerol in burley tobacco seedlings, indicating that glycerol application promoted nitrogen assimilation. Nitrate will be difficult to utilization once it is stored^37^. Nitrate transport was lower in burley tobacco than in other tobacco types^8^. *NPF3*.*1* and *NPF7*.*3* are low-affinity proton-dependent bidirectional nitrate transporter that are involved in regulation of nitrite uptake into chloroplasts and nitrate loading into xylem in higher plant^38^. Expression of genes *NPF3*.*1* and *NPF7*.*3* were up-regulated by spraying glycerol in burley tobacco seedlings, indicating that glycerol enhanced nitrate transport. These results showed that glycerol could increase the ability of nitrate transport and nitrate assimilation in burley tobacco seedlings and decrease nitrate content under 20% reducing nitrogen application conditions. And 20% nitrogen reduction with spraying glycerol would be an effective approach of decreasing nitrate accumulation in burley tobacco.

In conclusion, spraying glycerol decreased nitrate accumulation and promoted carbohydrates formation in burley tobacco seedlings at different nitrogen levels. Reducing nitrogen application inhibited the expression of genes involved in photosynthesis and reduced leaf biomass. By spraying glycerol, chlorophyll content, photosynthesis rate, total soluble sugar, reducing soluble sugar content and leaf biomass were all increased. Total nitrogen and nitrate contents were significantly decreased by glycerol application in burley tobacco seedlings under different nitrogen conditions (*P* < 0.01). Spraying glycerol under 20% less nitrogen application condition was an effective method to reduce nitrate accumulation in burley tobacco seedlings while maintaining same levels of leaf biomass. Glycerol triggered the biosynthesis of carbohydrates and promoted the abilities of nitrate reductase, nitrate assimilation and nitrate transport, leading to the increase of carbohydrates and to the reduction of nitrate in burley tobacco seedlings.

## Materials and Methods

### Plant materials

Seeds of burley tobacco varieties TN90 and TN86 were sown in a floating system in greenhouse that maintained a temperature ranging from 19 °C (night) to 28 °C (day), average photosynthetic photon flux density of 600 μmol m^−2^ s^−1^ and relative humidity 80%. Seedlings (sown 40 days later) were transplanted in 25 cm × 30 cm (diameter × depth) plastic pots (plant/pot) while seedlings had four to five permanent leaves. Seedlings were cultivated with Hoagland solution containing either 4 mM, 24 mM and 19.2 mM nitrogen levels^8^. All nutrient solutions were continuously aerated with an air pump. After germination, nutrient solution was replaced every six days. Nutrient solutions were refreshed every two days when seedlings were transplanted (sown 30 days later) in plastic pots. Seedlings were treated after three days (for recovery).

### Experiment 1

Deficient nitrogen level: (1) Nd-TN90, 4 mM, TN90; (2) Nd-TN86, 4 mM, TN86. Sufficient nitrogen level: (1) Ns-TN90, 24 mM, TN90; (2) Ns-TN86, 24 mM, TN86. Sampling and determining were carried out 10 days after seedlings being transplanted (around 10: 00 am). Leaves of five plants from each treatment were mixed and frozen in liquid nitrogen immediately, and then kept at −80 °C. Leaves of fifteen seedlings per treatment were deactivated at 105 °C for 20 min and then dried at 60 °C for 48 h and weighed, finally. Every treatment had three biological replicates.

### Experiment 2

Varieties of TN90 and TN86: Nitrogen reduction level, 20% less nitrogen application condition: (1) N80-CK, 19.2 mM nitrogen level, spraying pure water; (2) N80-Gl, 19.2 mM nitrogen level, spraying 0.1% glycerol. Nitrogen sufficiency level: (1) N100-CK, 24 mM nitrogen level, spraying pure water; (2) N100-Gl, 24 mM nitrogen level, spraying 0.1% glycerol. Every treatment had three biological replicates. In our preliminary research, seven glycerol concentrations of 0.00%, 0.025%, 0.05%, 0.075%, 0.1%, 0.15% and 0.2% were compared to test their effects on reducing nitrate content in burley tobacco, and 0.1% was proved to be the most effective concentration.

The two sides of all leaves were sprayed by glycerol with a manual pump at different nitrogen levels (around 8: 00–9: 00 am). Control seedlings were sprayed with same volume of pure water. Sampling and determination were carried out after seedlings being treated 2 days and 7 days (around 10: 00 am), respectively.

Leaves of five plants from each treatment were mixed and frozen in liquid nitrogen immediately, then kept at −80 °C. Fifteen plants each treatment were separated into root, stem and leaf and then deactivated at 105 °C for 20 min and dried at 60 °C for 48 h. Tissues (root, stem and leaf) were weighed and ground to pass through a screen with 60 meshes, and the final powder mixture was used to determine nitrate, total nitrogen, total soluble sugar and soluble reducing sugar content in plant.

Note: It was reported that glycerol inhibited cotyledon greening and development of true foliar leaves at 100 mM (0.728%, V/V)^11^. And the dose of glycerol (0.1%, V/V) in this work was much lower than the concentration of 100 mM.

### Measurement of pigment content and photosynthetic rate

Pigment content was determined by 95% ethanol^39^. Photosynthetic rate (Pn) was measured with a portable photosynthesis system (LI-COR Biotechnology, 6400XT, Lincoln, NE, USA)^40^.

### Measurement of carbonitride content

Nitrate content was determined by the method described by Cataldo^41^. Soluble protein content was assayed according to Li^42^. Total nitrogen, total soluble sugar and reducing sugar content were determined according to methods modified from the Chinese Tobacco Industry standard (YC/T 161, 159-2002)^8^.

### RNA Extraction, Preparation of cDNA Library, and Sequencing

Total RNA was extracted using the mirVana miRNA Isolation Kit (Ambion) following the manufacturer’s protocol. RNA integrity was evaluated with the Agilent 2100 Bioanalyzer (Agilent Technologies, Santa Clara, CA, USA). Samples with RNA Integrity Number (RIN)≥7 were subjected to the subsequent analysis. TruSeq Stranded mRNA LT Sample Prep Kit (Illumina, San Diego, CA, USA) was applied to conduct the libraries by the manufacturer’s instructions. Then the libraries were built on the Illumina sequencing platform (HiSeqTM 2500 or Illumina HiSeq X Ten) and 125 bp/150 bp paired-end reads were generated. The quality control was evaluated on the remaining reads according to NGS QC Toolkit^43^. Low quality date was removed, and 92.86% of Q20 percentage from clean reads was mapped to reference P. trichocarpa genome (ftp://ftp.solgenomics.net/genomes/Nicotiana_tabacum/assembly/Ntab-K326_AWOJ-SS.fa.gz) using bowtie2 or Tophat (http://tophat.cbcb.umd.edu/)^44,45^.

### RNA-Seq analysis

Transcript profiles of RNA-seq were analyzed by calculating the read fragments per kilo base per million mapped reads (FPKM). FPKM value of each gene was calculated using cufflinks, and the read counts of each gene were obtained by htseq-count^46,47^. DEGs were identified using the DESeq (2012) functions estimate Size Factors and nbinom Test^48^. In the process of DEGs screening, fold change (FC)>2 or FC <0.5, *p*-value < 0.05, was used as threshold to determine the significance of gene expression differences between nitrogen deficiency condition and nitrogen sufficiency condition; moreover, genes of DEGs, both nitrogen sufficiency and nitrogen deficiency condition, with FPKM < 1 were removed. FC is radio of FPKM between nitrogen sufficiency and nitrogen deficiency conditions. Gene function was annotated based on databases of NR (NCBI non-redundant protein sequences), KOG (Clusters of Orthologous Groups of proteins)^49^, Swiss-Prot (A manually annotated and reviewed protein sequence database)^50^, KO (KEGG Ortholog database)^51^, GO (Gene Ontology)^52^. GO enrichment and KEGG pathway enrichment analysis of DEGs were respectively achieved using R based on the hypergeometric distribution. Heatmaps analysis of genes expression was generated with R (3.4.1 version) pheatmap package^53^.

### Gene expression analysis by qRT-PCR

Eight genes determined by qRT-PCR were randomly selected to validate the transcript levels obtained by RNA-Seq (Experiment 1). RT reactions were performed in a Gene Amp® PCR System 9700 (Applied Biosystems, Foster City, USA) and Gene Amp® PCR System 9700 (Applied Biosystems, Foster City, USA). Expression of twelve genes correlated with glycerol metabolism, carbohydrates biosynthesis and nitrogen metabolism were observed (Experiment 2). Real-time PCR was performed using Light Cycler® 480 II Real-time PCR Instrument (Roche, Basel, Swiss). Reactions were incubated in a 384-well optical plate (Roche, Basel, Swiss) at 95 °C for 5 min, followed by 40 cycles of 95 °C for 10 s, 60 °C for 30 s. *L25* was used as the endogenous control (Supplementary Tables S1, S2). The expression levels of mRNAs were normalized and calculated using the 2^−ΔΔCt^ method^54^.

### GC-MS analysis of metabolites

#### Metabolites Extraction

Five plants per treatment were determined by GC-MS. Take 60 ± 1 mg sample into the 2 mL EP tubes, extracted with 0.48 mL extraction liquid (V_Methanol_: V_H2O = _3: 1), add 20 μL of adonitol (0.5 mg mL^−1^ stock in dH_2_O) as internal standard, vortex mixing for 30 s; Homogenized in ball mill for 4 min at 45 Hz, then ultrasound treated for 5 min (incubated in ice water); Centrifuge for 15 min at 12000 rpm, 4 °C; Transfer the supernatant (0.4 mL) into a fresh 2 mL GC/MS glass vial. Dry completely in a vacuum concentrator without heating; Add 40 μL Methoxyamination hydrochloride (20 mg mL^−1^ in pyridine) incubated for 30 min at 80 °C; Add 60 μL of the BSTFA regent (1% TMCS, v/v) to the sample aliquots, incubated for 1.5 h at 70 °C; All samples were analyzed by gas chromatograph system coupled with a Pegasus HT time-of-flight mass spectrometer (GC-TOF-MS).

#### GC-TOF-MS Analysis

GC-TOF-MS analysis was performed using an Agilent 7890 gas chromatograph system coupled with a Pegasus HT time-of-flight mass spectrometer. The system utilized a DB-5MS capillary column coated with 5% diphenyl cross-linked with 95% dimethylpolysiloxane (30 m × 250 μm inner diameter, 0.25 μm film thickness; J&W Scientific, Folsom, CA, USA). A 1 μL aliquot of the analyte was injected in split less mode. Helium was used as the carrier gas, the front inlet purge flow was 3 mL min^−1^, and the gas flow rate through the column was 1 mL min^−1^. The initial temperature was kept at 50 °C for 1 min, then raised to 310 °C at a rate of 10 °C min^−1^, then kept for 8 min at 310 °C. The injection, transfer line, and ion source temperatures were 280, 280, and 250 °C, respectively. The energy was −70 eV in electron impact mode. The mass spectrometry data were acquired in full-scan mode with the m/z range of 50–500 at a rate of 20 spectra per second after a solvent delay of 6.27 min.

#### Data preprocessing and annotation

Chroma TOF 4.3 X software of LECO Corporation and LECO-Fiehn Rt x 5 database were used for raw peaks exacting, the data baselines filtering and calibration of the baseline, peak alignment, deconvolution analysis, peak identification and integration of the peak area^55^. Both of mass spectrum match and retention index match were considered in metabolites identification.

### Statistical analysis

Figures were applied with Origin Pro 9.0 (Origin Lab Corporation, Northampton, MA, USA). Heatmaps were generated with R (3.4.1 version) pheatmap package. Statistical analysis was performed with SPSS 20 software (IBM, Palo Alto, CA, USA). For comparison between two data sets, a Student’s t test was used. For analysis of three or more sets of data, individual comparisons between mean values performed by using the least significant differences (LSD) test. *P < 0.05, **P < 0.01 were considered statistically significant.

## Electronic supplementary material

Supporting tables

## Data Availability

All of the materials, data and associated protocols will be made available upon request without preconditions. All data generated or analyzed during this study are included in this published article (and its Supplemental Information files).
