# Supplementary material for: Metabolome and molecular basis for carbohydrate increase and nitrate reduction in burley tobacco seedlings by glycerol through upregulating carbon and nitrogen metabolism
Source: Sci Rep. 2018 Sep 5;8:13300. doi: 10.1038/s41598-018-31432-3 (PMC6125293; doi:10.1038/s41598-018-31432-3)
Supplement: Supplementary file 1 — Supporting tables [file 41598_2018_31432_MOESM1_ESM.pdf]

Metabolome and molecular basis for carbohydrate increase and nitrate reduction in burley tobacco seedlings by glycerol through upregulating carbon and nitrogen metabolism

Yafei Li [1 †], Dong Chang [1, 2 †], Huijuan Yang [1], Jing Wang [1], Hongzhi Shi [1 *]
1 National Tobacco Cultivation & Physiology & Biochemistry Research Center, Henan Agricultural University, 450002, Zhengzhou, China; 2 Pingdingshan Branch of Henan Provincial Tobacco Company, Pingdingshan, 467002, Pingdingshan, China

***Corresponding author:** Hongzhi Shi: shihongzhi88@163.com; Tel: +86-136-1383-0281

Author information:
Yafei Li: liyafei@henau.edu.cn
Dong Chang: cd411@outlook.com
Huijuan Yang: huijuan@zju.edu.cn
Jing Wang: wj2009g@163.com

† These authors contributed equally to this work.

Supporting Table S1. The primers used for qRT-PCR in Experiment 1

No.	Gene Symbol	Forward primer	Reverse primer	Product length(bp)	Ta(℃)
1	WRKY70	TGCGAAGCAAACAAACAAGT	ATGTGTGATGACCATAGTACG	79	60
2	TIFY10A	AGAATTACTTCAACTGCACCA	CTAATCCAAGCCATGCCTTAT	88	60
3	EXO	CATTCCACCAGCCCATTTAC	CATACCATCAACACCCACAT	77	60
4	XTH22	GATGATTGGGCTACAAGAGG	GCACTAAAGGGTGCTTTACT	62	60
5	MYB44	GCGGAGGTGAGGAACTAT	TTGCTGAAACTGCTGTTGTC	96	60
6	PGK	AAGCCACTTGTACCAAGAC	CCTCCTTCTGGTATTTCGG	110	60
7	PPC16	ATGTCCTTGATGCCATTACC	GTTCCTCAGACCACTCTCG	63	60
8	EGY1	TGCTACCAGTTGGATGTCTTG	AAGCCCAGAAGTGTGTATG	103	60
9	L25	CCCCTCACCACAGAGTCTGC	AAGGGTGTTGTTGTCCTCAATCTT	51	60

Supporting Table S2. The primers used for qRT-PCR in Experiment 2

No.	Gene Symbol	Forward primer	Reverse primer	Product length(bp)	Ta(°C)
1	PGK	AAGCCACTTGTACCAAGAC	CCTCCTTCTGGTATTTCGG	110	60
2	GLPK	TGTTCAGTGGCTTAGGGACA	GATGCCAACTCCTCGATTTC	66	60
3	NIA1	TTAGGGTCATTTCTCGACGTG	GAAATTGCCCTTTCCTTGGTA	66	60
4	SPS	TCCATTGAGAGAGCTTGCTAA	AGGAGGACTGATGAATTTGTG	90	60
5	SUS2-2	TGAGATGCTCAAGCGCATA	CAGGCAGCAACCGAGTAA	77	60
6	NPF3.1	AGAACAGAGAGTTGGATGCTA	TGTCATCACCATCAACGACC	105	60
7	NLP7	GCTCAGACATGGCTACCT	AGCTCTGAACTCATAGCCT	105	60
8	gpmA	TATCTTCACAAGCCCATCCT	ATCCAGAGTTACCATTACTTCG	117	60
9	NPF7.3	CTCTTTGTTGAGCAAGGTGA	ATATGACGCCTGCTACACTAA	106	60
10	PPC16	ATGTCCTTGATGCCATTACC	GTTCCTCAGACCACTCTCG	63	60
11	EGY1	TGCTACCAGTTGGATGTCTTG	AAGCCCAGAAGTGTGTATG	103	60
12	CP12-2	GATGTGGTTGAAGAGGCG	CAGTCTCAGGATTATCCTTGC	103	60
13	L25	CCCCTCACCACAGAGTCTGC	AAGGGTGTTGTTGTCCTCAATCTT	51	60
